# Anticancer Drug Discovery Based on Natural Products: From Computational Approaches to Clinical Studies

**DOI:** 10.3390/cancers17152507

**Published:** 2025-07-30

**Authors:** Rajeev K. Singla, Anupam Bishayee

**Affiliations:** 1 Department of Pharmacy and Institutes for Systems Genetics, Center for High Altitude Medicine, Frontiers Science Center for Disease-related Molecular Network, West China Hospital, Sichuan University, Chengdu 610041, China; 2 School of Pharmaceutical Sciences, Lovely Professional University, Phagwara 144 411, Punjab, India; 3 Department of Pharmacology, College of Osteopathic Medicine, Lake Erie College of Osteopathic Medicine, Bradenton, FL 34211, USA

## 1. Introduction

**Figure 1:**
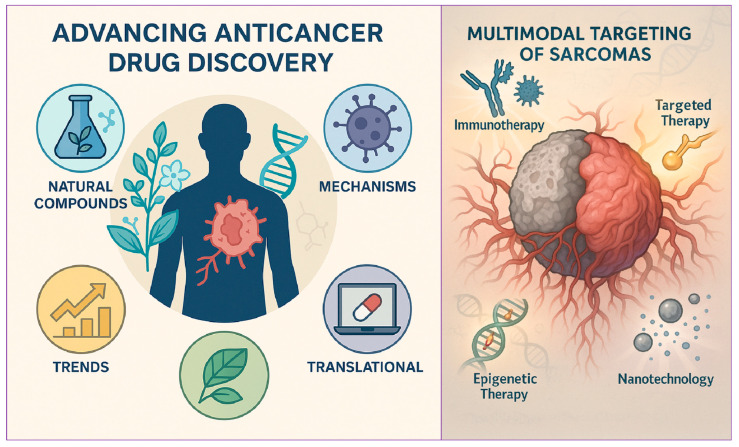
Multimodal strategies must be adopted while targeting various stages of cancers. Figure was generated with assistance of large-language models (LLMs) ChatGPT 4o, OpenAI’s DALL·E, and Deepseek.

Cancer represents a major public health, societal, and economic challenges in the 21st century. According to the data published by the International Agency for Research on Cancer/GLOBOCAN, close to 20 million new cases of cancer and 9.7 million deaths from this disease occurred in 2022 [1]. Furthermore, it has been estimated that approximately one in five men or women develop cancer in their lifetime [1]. In the United States alone, more than 2 million new cancer cases and 618,120 cancer deaths are expected to occur in 2025 [2]. All these data provide a strong impetus to explore novel agents for the prevention and treatment of cancer. Natural products have been an expansive source of new anticancer drugs. During the last several decades, a plethora of natural agents, especially bioactive phytocompounds, have been investigated based on cell culture assays, animal tumor models, and human subjects, to understand their potential for cancer prevention and treatment [3]. Figure 1 illustrates the multimodal target-based advancement required for anticancer drug discovery. Moreover, recent developments in cheminformatics and bioinformatics—such as network-pharmacology-based studies, pharmacokinetics and toxicity prediction, molecular docking, molecular dynamics simulations, artificial intelligence (AI)- and machine learning (ML)-based models—have contributed to accelerating anticancer drug development. Considering such progress, we are pleased to introduce this Special Issue, which documents recent advances in our knowledge of cancer preventive and the therapeutic efficacy of natural substances, with a view to understanding cellular and molecular mechanisms of action.

## 2. An Overview of Published Articles

This thematic issue contains five original research papers, four narrative reviews and one systematic review article which capture the translational aspects of preclinical anticancer studies to clinical development.

Gupta and colleagues (Contribution 1) investigated the cytotoxic activity of the mechanisms of action of two bioactive triterpenoids, namely oleanolic acid and ursolic acid, individually and in combination using MCF-7 (estrogen receptor-positive) and MDA-MB-231 (triple-negative) breast cancer cells. The results of this study indicated that both phytocompounds were equally effective against two cell lines. Interestingly, a combination of phytochemicals was more effective than ursolic acid alone. Mechanistic studies revealed that the phytochemical combination induced excessive autophagy via inhibition of phosphoinositide 3-kinase (phosphoinositide 3-kinase)-mediated phosphorylation of Akt and mammalian target of rapamycin (mTOR). These results underscore the potential of the tested natural compounds for cancer therapy, especially in adjuvant or neoadjuvant settings.

The study of Campanelli et al. (Contribution 2) established a genetically engineered mouse model which mimics advanced prostate cancer due to overexpression of prostate-specific metastasis-associated protein 1 (MTA1) and a loss of phosphatase and tensin homolog (PTEN) expression. Utilizing this tumor model, the authors showed that gnetin C, a stilbene family polyphenol, suppressed abnormal cell proliferation and angiogenesis and promoted apoptosis through efficient targeting of the MTA1/PTEN/Akt/mTOR pathway. This study provides a “proof-of-principle” that a novel natural compound, such as gnetin C, can target specific oncogenic signaling pathways for clinical management of advanced prostate cancer.

Mohamed and coinvestigators (Contribution 3) conducted a study to evaluate possible anticancer activities of naringin, a polyphenolic phytochemical, and naringin-dextrin nanocomposites (Nar-Dx-NCs) against diethylnitrosamine (DENA)/2-acetylaminofluorene (2-AAF)-induced lung carcinogenesis in rats. Naringin’s chemopreventive action against the DEN/2-AAF lung tumor model was amplified by Nar-Dx-NCs as this novel formulation diminished lung carcinogenesis by decreasing tumor cell proliferation, activating apoptosis, and suppressing oxidative stress and inflammation. The encouraging results of this study may lead to additional research to understand the full potential of nanocomposites of naringin again human lung cancer.

Awad et al. (Contribution 4) investigated the therapeutic effect of crocin, a bioactive compound present in saffron, and its combination with sorafenib, a chemotherapeutic drug for hepatocellular carcinoma (HCC), against DENA-induced rat liver carcinogenesis. It was found that crocetin potentiated the anticancer effect of sorafenib. Further, a combined therapy also caused the highest level of apoptosis and a reduction in proliferation, as well as in β-catenin, cyclooxygenase, and nuclear factor-κB levels in tumor tissue, compared to monotreatment with each agent. These encouraging results present a potential strategy to optimize the anti-HCC effect of sorafenib by using a natural compound.

Boulos et al. (Contribution 5) tested the hypothesis that targeting c-MYC, a proto-oncogene, using adapalene, a third-generation retinoid, represents a novel treatment for hematological malignancies. Adapalene exhibited a potent cytotoxic effect through c-MYC inhibition, tubulin network suppression, and DNA damage in vitro and suppressed tumor growth via apoptotic and autophagic cell death in a xenograft zebrafish tumor model. The significance of this work lies in the fact that an adapalene-based design may lead to more effective and targeted therapies for multiple myeloma.

In this collection, four review articles present recent research developments on cancer prevention and anticancer therapeutic activities of various plant-based products and phytochemicals.

Pawłowski and colleagues (Contribution 6) have reviewed the potential anticancer properties of natural polyphenols, specifically curcumin, resveratrol, epigallocatechin-3-gallate, genistein, and quercetin, and related mechanisms of action, such as the inhibition of type IV collagenase enzymes, namely matrix metalloproteinase (MMP)-2 and MMP-9. Emerging evidence based on in vivo and in vitro studies highlighted the polyphenol’s antioxidant, anti-inflammatory, pro-apoptotic, antiangiogenic, anti-invasive, and antimetastatic properties. Interestingly, authors have also highlighted various limitations, such as their low solubility and bioavailability, and suggested the utilization of advanced drug delivery systems to overcome these issues.

Jiménez-González and colleagues (Contribution 7) have focused on the anticancer potential of crude extracts and isolated bioactive compounds of the plants belonging to the *Euphorbiaceae* family. They have also covered nanoformulations of these natural products. Potential anticancer molecular mechanisms, such as the induction of apoptosis, modulation of oxidative stress, and interference with cancer cell signaling, highlighted the promising translational potential of *Euphorbiaceae*-derived bioactive compounds for future drug development.

Choudhary and colleagues (Contribution 8) have comprehensively reviewed the major contributing signaling pathways involved in the pathophysiology of lung cancer, currently available treatment options, and the anti-lung cancer potential of naturally occurring bioactive agents, such as alkaloids, terpenoids, phenolics, and sulfur-containing compounds. These phytochemicals were found to target important signaling cascades, including RAF/mitogen-activated protein kinase kinase (MEK)/extracellular signal-regulated kinase (ERK), PI3K/Akt/mTOR, and Janus kinase/signal transducer and activator of transcription (JAK/STAT). Credible evidence based on in vitro, in vivo, and emerging clinical data highlighted the possible translational potential of various natural compounds.

Another comprehensive review article, by Pal and colleagues (Contribution 9), focused on histone deacetylase inhibitors (HDACis), both natural as well as synthetic compounds, for the treatment and management of hematological malignancies, such as acute myeloid leukemia, B-cell lymphoma, and multiple myeloma. The modulation of histone acetylation results in gene expression associated oxidative stress, DNA damage, apoptosis, and autophagy; these are possible mechanisms underlying the anticancer potential of HDACis, with enhanced outcomes, particularly in combination therapies.

In their systematic review and meta-analysis, Odeniran and colleagues (Contribution 10) evaluated the efficacy and safety of camptothecin-derived chemotherapeutics, such as irinotecan and topotecan, combined with other chemotherapeutic agents based on phase II and III clinical trials. This work covered various cancers, including lung, esophageal, gastric and colorectal cancers. It has been observed that irinotecan in combination with cisplatin could significantly improve response rate and progression-free survival in non-small cell lung cancer. However, irinotecan in combination with bevacizumab yielded superior outcomes in colorectal cancer.

**Figure 2:**
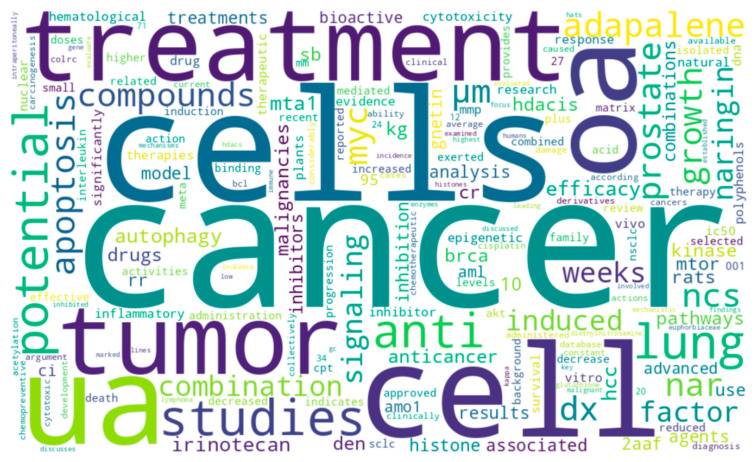
Word cloud with frequency mapping for important cancer and natural product terminology in the abstracts of these papers. This figure was generated with assistance of large-language models (LLMs) ChatGPT 4o, OpenAI’s DALL·E, and Deepseek.

Figure 2 illustrates the frequency mapping of various terminologies presented in the abstracts of the 10 articles of this Special Issue. Various frequently occurring terms were apoptosis, HDACis, naringin, MTA1, adapalene, mTOR, 2-AAF, among others. These articles featured a wide range of information where natural products have been evaluated and targeted against various kind of cancer, including but not limited to breast cancer, prostate cancer, lung cancer, hematological cancers, colorectal cancer, gastric cancer, and hepatocellular carcinoma. All these studies indicate that PI3K/Akt/mTOR, NF-κB, and MMP-related ECM pathways are the most commonly targeted pathways. Moreover, c-MYC, TP53, HDACs, and Bcl-2/Bax were the most studied gene targets in these studies. Gnetin C, oleanolic acid, ursolic acid, naringin, adapalene, and crocin were the promising translational natural agents, discussed repeatedly in these papers. Furthermore, cytotoxic autophagy, natural HDAC inhibitors, and bioavailability-improving nanotechnologies are emerging areas of focus.

Historically, natural product-based drug discovery has been hindered by many concerns, involving a lack of systematic evaluations, unclear molecular targets and signaling networks, translational barriers, and limited synergy with modern tools. The articles published in our Special Issue tackle some of these concerns by conducting comprehensive meta-analyses, elucidating molecular mechanisms, utilizing advanced in silico and 3D modeling approaches, and exploring combination therapies.

## 3. Future Directions and Perspectives

Although this Special Issue advances knowledge in the domain of natural product-based anticancer drug discovery, the field is growing at a fast pace. Some of the avenues which remains underexplored and where researchers can place more focus in the future are thus listed below:

AI-guided compound screening: AI and ML can rationally expedite natural product screening by filtering out large datasets, such as PubChem, based on the prediction of efficacy, synergy, and toxicity [4,5].

Personalized medicine and biomarker integration: Focus should be on the genomic/molecular stratification to guide natural product-based therapies [6].

A. Emerging trends of natural products in immunotherapy and resistance: Exploration of natural products influencing immune checkpoints, tumor microenvironment, and host–tumor interactions [7,8].

B. Overcoming limitations of natural products: Natural products are not devoid of limitations, and limitations, such as bioavailability and supply, along with other factors limit their translational potential [9,10,11].

C. Clinical translation pathways: Focused studies with concerted efforts in pharmacokinetics, toxicology, and formulation optimization, so as to bridge gap between pre-clinical and clinical utility [12,13].

D. Microbiome and cancer: Role of microbiota in cancer development, progression, and therapeutic response [14,15].

## 4. Conclusions

In conclusion, this Special Issue highlights the growing significance of natural products as potential anticancer agents. Through the publication of a collection of original articles and comprehensive reviews, we underscore the therapeutic values and translational potential of bioactive phytochemicals. We urge continued multidisciplinary research and collaboration in advancing modern oncology. Finally, we would like to thank all the editorial team members, authors, and the reviewers for contributing to this Special Issue and advancing anticancer drug discovery and development based on natural products.
